# SOS – save our seaside! The microbiological risks to human health of raw sewage in our coastal waters

**DOI:** 10.1099/mic.0.001529

**Published:** 2025-02-07

**Authors:** Jonathan A. G. Cox

**Affiliations:** 1 College of Health and Life Sciences, Aston University, Birmingham, UK

**Keywords:** coastal water contamination, public health, sewage

## Abstract

Most of us enjoy a day at the beach, but we rarely consider that recreational use of our coastal waters could impact our health. This article explores the microbiological threats of sewage discharge to our fun in the sea and proposes a simple way to make sure it’s only sand that you and your family bring home from a visit to the seaside.

## Introduction

Despite having spent the last 18 years living in the land-locked midlands, I remain a card-carrying thalassophile (to save you from looking it up, a *thalassophile* is someone who loves the sea). I grew up in Plymouth, Devon, and spent much of my childhood in, on or around the sea before moving to Birmingham for university in 2007. Although the decision to relocate to the midlands has taken me on an incredible scientific voyage, I’ve always missed the sea – canals just don’t cut it! Fortunately, having family in the southwest has always given me just cause to regularly return to the coast to satisfy my cravings for the sea. Despite rapidly approaching my fortieth anniversary of birth, I still sail, sea swim, paddleboard, kayak and surf (badly) at every given opportunity. However, my addiction to the sea is not without consequences. In spring 2024, whilst sea swimming, I contracted a bilateral atypical bacterial pneumonia that completely knocked me off my feet. I was lucky, and the bacteria causing my infection were sensitive to clarithromycin. Following my illness, I discovered Surfers Against Sewage [1] and their report of raw sewage being dumped close to where I had been swimming hours before I took to the water. The experience got me thinking about the bacterial pathogens in our beautiful coastal waters and the risks they pose to those like me, mad enough to take to the sea all year round.

My investigation has led me to understand that the pollution of coastal seawater by sewage has become an increasingly critical issue for public health, particularly in the UK. Pathogenic bacteria found in sewage are of significant concern as they can directly affect the quality of recreational waters and overall public health [2]. In this article, I investigate the major pathogenic bacteria associated with sewage contamination in UK coastal waters, their potential risks and the measures taken to mitigate their impact. As with most things these days, money seems to be the limiting factor, and the investment required to improve our national infrastructure to decontaminate our waste before releasing it into our coastal waters seems, for the time being at least, to be dead in the water.

## Pathogenic bacteria in sewage

Sewage contamination in coastal waters can introduce a variety of harmful micro-organisms, including pathogenic bacteria that can cause gastrointestinal, respiratory and skin infections. Among the bacterial pathogens found in sewage-contaminated seawater are *Brucella* spp., *Chlamydia* spp., *Escherichia coli* (enteropathogenic antibiotic-resistant strains), *Leptospira* spp., *Rickettsia* spp., *Salmonella* spp., *Treponema hyodysenteriae*, *Bacillus anthracis*, *Erysipelothrix rhusiopathiae*, *Mycobacterium* spp. and faecal *Streptococci* spp. The list goes on. These bacteria can be found in the intestinal tracts of humans and animals and can be transmitted through the ingestion, inhalation or contact with contaminated water [3].

*E. coli* is the most commonly studied bacterium in sewage-contaminated waters and is widely used as an indicator of faecal contamination [4]. High concentrations of *E. coli* (>1000 c.f.u./100 ml) in seawater often indicate the presence of other pathogenic micro-organisms, which may include *Salmonella*, *Campylobacter* and *Vibrio* species. These organisms that are common causes of foodborne illnesses are detected in sewage-contaminated waters [5].

## Risks to public health

The risks posed by pathogenic bacteria from sewage contamination are significant for both public health and marine ecosystems. The ingestion of contaminated water has led to outbreaks of gastroenteritis, with symptoms including diarrhoea, vomiting and fever [5]. For example, *Vibrio* infections, particularly those caused by *Vibrio parahaemolyticus* and *Vibrio vulnificus*, can cause severe shellfish-derived food poisoning and, in some cases, death. Concerningly, there is an increasing prevalence of these *Vibrio* spp. identified in UK coastal waters as sewage has been found to promote the growth of these pathogens [6,7]. Vulnerable populations, such as the elderly, immunocompromised individuals and pregnant women, are especially at risk.

Inhalation of sewage aerosol has resulted in outbreaks of bacterial pneumonia, and a study found that 58% of seawater drowning-associated pneumonia is caused by aerobic Gram-negative bacilli, some displaying levels of antibiotic resistance [3,8]. Whilst the direct causative data are sparce, on the balance of probabilities, inhaling sewage-contaminated seawater is likely to cause bacterial pneumonia.

Infections caused by exposure to sewage-contaminated seawater are not limited to gastrointestinal or respiratory. Many other types of bacterial infection have also been linked to faecal pathogens in our coastal waters, including skin and soft tissue infections, ear and eye infections and tonsilitis.

In addition to the direct human health risks, pathogenic bacteria can disrupt marine ecosystems. Sewage pollution can lead to algal blooms in coastal waters, which can harm marine life and reduce biodiversity. Pathogens from sewage are also impacting shellfish populations, which are filter feeders and, as a result, can accumulate harmful bacteria that may re-enter the human food chain, perpetuating antimicrobial resistance.

## Who’s in charge of the discharge?

To address the risks posed by sewage contamination, several regulatory and technological measures are in place. In the UK, the Environment Agency and DEFRA are responsible for regulating water quality through the Bathing Water Directive and the Shellfish Hygiene Directive, which set standards for microbial water quality at recreational beaches and in areas where shellfish are harvested. Monitoring programs are in place to test for pathogens such as *E. coli* and *Enterococci*, and sewage treatment plants are required to meet strict discharge standards to minimize the release of harmful bacteria into the environment. Storm overflows were intended to release surplus sewage into the sea on rare occasions, but despite this intention, some water companies are responsible for up to 200 discharges of raw untreated sewage into our coastal waters each year [2].

Whilst new sewage treatment technologies, such as tertiary filtration and UV disinfection, have been developed to reduce bacterial concentrations in effluent before it is discharged into coastal waters, they are expensive to implement and are limited by the volume of sewage. During heavy rainfall events, water companies will continue to discharge untreated sewage into the sea, posing a direct threat to water quality and public health.

## Conclusion

The presence of pathogenic bacteria in sewage-contaminated seawater in the UK is a significant public health concern. Pathogens like *E. coli*, *Salmonella*, *Vibrio* and *Campylobacter*, to name but a few, can lead to severe infections and perpetuate the spread of antibiotic resistance. Whilst regulatory measures and advanced sewage treatment technologies are promised, ongoing vigilance and investment in infrastructure are essential to mitigate the risks posed by sewage pollution and to protect both public health and our coastal waters.

## Final thoughts of a thallasophile

This makes for pretty sober reading, and it is clear that, when taking to the sea, we should be less concerned about what’s lurking beneath the surface and more concerned about what lies within. Am I going to stop enjoying the water? No. However, armed with this new knowledge and the Safer Seas and Rivers Service App run by Surfers Against Sewage, I will always check to see if sewage has been discharged in the area before taking to the water, especially with my children. Professor Whitty, of ‘*next slide please*’ fame, said himself in a report from Department of Health and Social Care regarding sewage in water, ‘*Nobody wants a child to ingest human faeces*’. You’re not wrong there, Chris. Whilst we’re waiting for the improved management, innovation and investment that is required to solve the issue and save our seaside, don’t bury your head in the sand regarding water quality. Check before you swim. Afterall, prevention is always better than cure.
